# First person – Casey Griffin

**DOI:** 10.1242/dmm.052355

**Published:** 2025-03-24

## Abstract

First Person is a series of interviews with the first authors of a selection of papers published in Disease Models & Mechanisms, helping researchers promote themselves alongside their papers. Casey Griffin is first author on ‘ Deletion of *sf3b4* causes splicing defects and gene dysregulation that disrupt craniofacial development and survival’, published in DMM. Casey is an Associate Research Scientist in the lab of Jean-Pierre Saint-Jeannet at the College of Dentistry, investigating the role of neural crest cells during the development of the face.


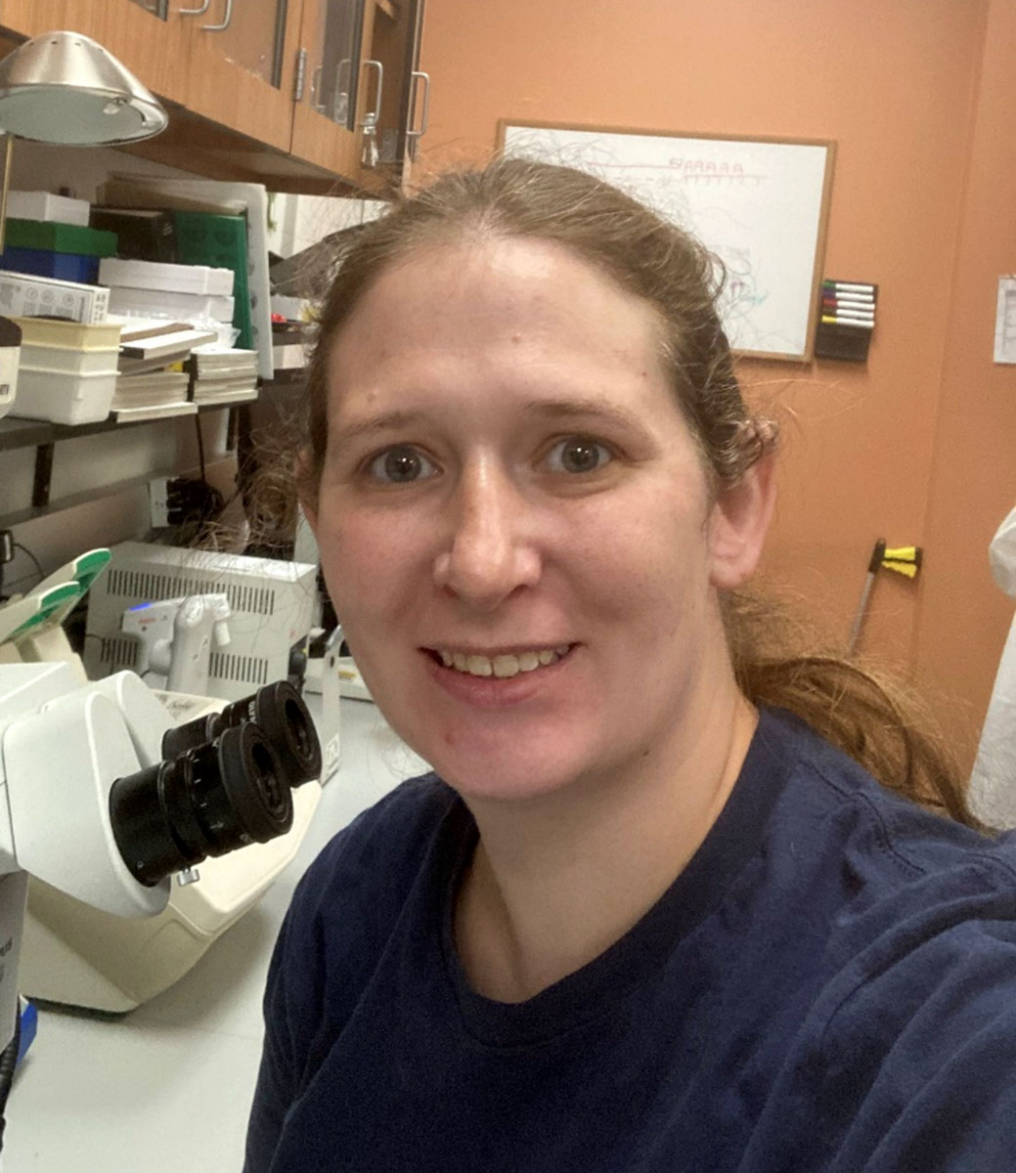


**Casey Griffin**

**Who or what inspired you to become a scientist?**

My father is a scientist, a biochemist, so I grew up always surrounded by science and learning about the life of a scientist. However, I probably wouldn't have become a scientist without the help of ‘Bill Nye the Science Guy’ and ‘The Magic School Bus’. I loved watching those shows and reading those books as a kid, and it made me dream of becoming an astronaut or a paleontologist or a doctor. It wasn't until I was in high school and taking biology classes that I knew for sure I wanted to be a scientist.

[…] why do craniofacial spliceosomopathies have such tissue-specific phenotypes when the mutations are in ubiquitously expressed genes?

**What is the main question or challenge in disease biology you are addressing in this paper? How did you go about investigating your question or challenge?**

The overarching question we have to ask is why do craniofacial spliceosomopathies have such tissue-specific phenotypes when the mutations are in ubiquitously expressed genes? To understand this, we study Nager syndrome and, as described in our paper, established a vertebrate system to study the loss of SF3B4 in the context of craniofacial development. This system allows us to begin to tease apart the underlying mechanisms of Nager syndrome by using expression analysis, RNA-sequencing and examination of splicing events. Our study resulted in a long list of candidate genes and pathways to examine in future studies.

**How would you explain the main findings of your paper to non-scientific family and friends?**

First, we developed a model of a disease called Nager syndrome, in which the face is underdeveloped. This model is in frogs who are missing a gene, causing the cells that give rise to the face to be underdeveloped or absent. We then used this model system to understand why disruptions in a specific gene give rise to Nager syndrome. We looked at all the genes expressed in the embryos of our model system, and can now use these data to begin to understand the mechanism underlying Nager syndrome.

**What are the potential implications of these results for disease biology and the possible impact on patients?**

Understanding the mechanisms of craniofacial spliceosomopathies will help to understand how the disorders unfold and manifest. This is beneficial for understanding a disease as a whole and extrapolating this information to the overall development of the face. While these types of disorder are difficult to discuss in terms of cures − because they begin in the womb during early development − understanding the underlying mechanisms of these disorders can help with more targeted patient care, improved quality of life and improved treatments for these disorders.

**Figure:**
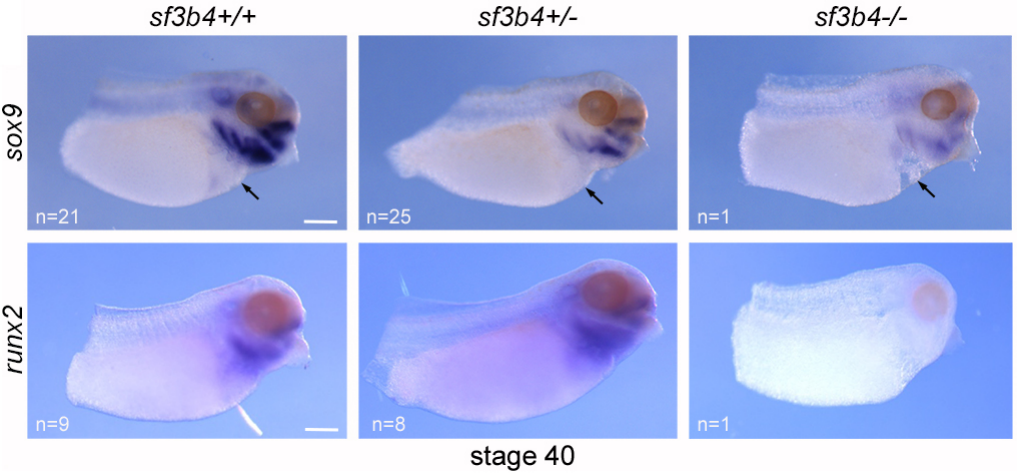
Expression of *sox9* marking craniofacial cartilage precursors in embryos with differing levels of *sf3b4* expression, showing a reduction in cartilage with a reduction of sf3b4.

**Why did you choose DMM for your paper?**

Our paper discusses a new animal model for a disease, and the aim of using that model is to understand the underlying mechanisms of the disorder. DMM highlights both aspects, with the goal of publishing and supporting new model systems of a disease. This seemed like the perfect fit for our paper.

It can be very demanding to have those personal and professional pressures going against each other

**Given your current role, what challenges do you face and what changes could improve the professional lives of other scientists in this role?**

I think the biggest challenge as a postdoc is the work-life balance. Many postdocs are in the stage of their live where they are settling down, starting families, but also in the stride of their career, making steps towards independence in research. It can be very demanding to have those personal and professional pressures going against each other. For me personally, I got pregnant during my postdoc, so I had to balance work with a lot of life changes. My daughter is now three, and I feel like I've found a good place where I can get my work done and feel accomplished, but I also don't sacrifice too much time of being with my husband and daughter. It helps to have an understanding and supportive mentor, who understands what I am striving for both professionally and personally. Having more supportive mentors would definitely help postdocs across the board. Having PIs who are trained in mentorship would be really good, and having open communication is key to that relationship.

**What's next for you?**

I recently received a K99 award from the NIH/NIDCR, so I'm starting work on my fellowship project, which is focused on further understanding the underlying mechanisms of craniofacial spliceosomopathies by using frogs and stem cells as model systems. Other than that, I will be applying to faculty positions, hoping to start my independent lab in the next year or two.

**Tell us something interesting about yourself that wouldn't be on your CV**

I am an avid collector of Lego. I have more sets than I would care to admit but, lately, my focus has been on Star Wars sets. My dream set is the Mount Doom and Sauron's Eye set from the Lord of the Rings. I just love building the sets and seeing these iconic pieces in the flesh. My daughter has gotten into it too, helping me build the sets, and it's a great time for us to bond over something fun and engaging. One day soon maybe she will start adding to my collection with her own sets!
